# Personal construct therapy vs. cognitive behavioural therapy in the treatment of depression in women with fibromyalgia: a multicentre randomized controlled trial with a 6-month follow-up

**DOI:** 10.1192/j.eurpsy.2022.660

**Published:** 2022-09-01

**Authors:** M. Aguilera, C. Paz, M. Salla, G. Feixas

**Affiliations:** 1 Universitat de Barcelona, Department Of Cognition, Development And Educational Psychology, BARCELONA, Spain; 2 Universidad de Las Américas, School Of Psychology, Quito, Ecuador; 3 Universitat de Barcelona, Department Of Clinical Psychology And Psychobiology, BARCELONA, Spain

**Keywords:** Fibromyalgia Impact, Treatment Efficacy, Psychotherapy, RCT

## Abstract

**Introduction:**

Depressive symptoms are common in patients with Fibromyalgia (FM), a chronic and disabling pain syndrome. Psychological interventions are mostly focused in negative thinking and behavioural activation. However, several studies suggest that personal identity is also affected by FM.

**Objectives:**

We aimed to examine the effects of Personal Construct Therapy (PCT), an idiographic approach that emphasizes identity features and interpersonal construal, on depressive symptoms in women with FM.

**Methods:**

In the context of a multicentre parallel randomized trial (Trial Registry: NCT02711020), 106 women with FM and presenting depressive symptoms were randomized either to either Cognitive Behavioural Therapy (CBT; *n* = 55), taken as a gold standard comparison, or PCT (*n* = 51). In total, 69 patients completed the treatment and the six-month follow-up assessment (CBT = 32 and PCT = 37). Both treatments were applied on case formulation premises.

**Results:**

Linear mixed-effects models were performed to compare depressive symptoms between treatment conditions. Anxiety and pain measures were treated as secondary outcomes. Participants in both conditions significantly reduced their levels of depression and anxiety as well as the impact of FM but no significant between treatment differences were found. Analysis of clinically significant change for depressive symptoms and pain was also similar between both conditions.

**Conclusions:**

PCT resulted equally effective in the treatment of depressive symptoms in women with FM when compared with CBT, both offered in a modular format. Thus, PCT with tis focus on identity issues can be considered as an alternative treatment for these patients.

**Disclosure:**

No significant relationships.

